# Targeting of a Transporter to the Outer Apicoplast Membrane in the Human Malaria Parasite *Plasmodium falciparum*

**DOI:** 10.1371/journal.pone.0159603

**Published:** 2016-07-21

**Authors:** Liting Lim, Claire P. Sayers, Christopher D. Goodman, Geoffrey I. McFadden

**Affiliations:** School of BioSciences, University of Melbourne, Melbourne, Victoria, Australia; Bernhard Nocht Institute for Tropical Medicine, GERMANY

## Abstract

Apicoplasts are vestigial plastids in apicomplexan parasites like *Plasmodium*, the causative agent of malaria. Apicomplexan parasites are dependant on their apicoplasts for synthesis of various molecules that they are unable to scavenge in sufficient quantity from their host, which makes apicoplasts attractive drug targets. Proteins known as plastid phosphate translocators (pPTs) are embedded in the outer apicoplast membrane and are responsible for the import of carbon, energy and reducing power to drive anabolic synthesis in the organelle. We investigated how a pPT is targeted into the outer apicoplast membrane of the human malaria parasite *P*. *falciparum*. We showed that a transmembrane domain is likely to act as a recessed signal anchor to direct the protein into the endomembrane system, and that a tyrosine in the cytosolic N-terminus of the protein is essential for targeting, but one or more, as yet unidentified, factors are also essential to direct the protein into the outer apicoplast membrane.

## Introduction

The apicoplast is a relic plastid in apicomplexan parasites such as *Toxoplasma gondii*, the causative agent of toxoplasmosis, and *Plasmodium* spp., the causative agents of malaria [1, 2]. Apicoplasts are homologues of plastids (often known as chloroplasts) in algae and plants but apparently lost photosynthesis early in the development of Apicomplexa [3]. The organelle is indispensable [4, 5] and synthesises isoprenoid precursors, fatty acids and haem [6], which are essential to the parasites at certain stages of their life cycle [1, 2]. The essentially prokaryotic nature of apicoplasts, which like plastids and chloroplasts ultimately derive from bacterial endosymbionts, has made them attractive as drug targets. Commonly used antimalarials such as doxycycline, rifampicin and clindamycin perturb apicoplast function and kill parasites [7, 8].

Much of our understanding of how apicoplasts work came through predicting their proteome on the basis of understanding how nucleus-encoded proteins are targeted into the organelle across its four bounding membranes [6, 9, 10]. Approximately 400 *Plasmodium falciparum* proteins have a bipartite N-terminal leader [9, 10] that is sufficient and necessary for targeting a protein into the apicoplast stroma [11] or the inner membrane [12]. The first element of the bipartite leader is a standard signal peptide [13] that orchestrates co-translational insertion of the protein into the rough endoplasmic reticulum (ER) [11, 14]. On entering the ER lumen, the signal peptide is cleaved off, probably by a signal peptidase [15]. Signal peptide cleavage exposes a new N-terminus, revealing a transit peptide with similar characteristics to those found in plants and alga [13]. The protein is then trafficked through the endomembrane system, via the Golgi [16], until an endomembrane vesicle carrying an apicoplast protein docks with the outer apicoplast membrane and deposits the protein in the space between the outer membrane and the next membrane in, namely the periplastid membrane [17]. In apicoplasts, the transit peptide is essential for traffic of proteins across the inner three bounding membranes and into the stroma, via a series of translocons [18–20]. Inner apicoplast membrane transporters also appear to use this cleavable, bipartite leader targeting [12].

The outer membrane of the apicoplast is the interface between the organelle and the parasite cytosol and is thus a critical boundary between the two compartments. We identified the first protein in this membrane and showed, unexpectedly, that it lacked a canonical bipartite leader [12]. An orthologue was later shown to also occur in the outer membrane(s) of *T*. *gondii* apicoplasts [21]. These two outer apicoplast membrane proteins belong to a family of translocators from the plastids of plants and algae known as the plastid phosphate translocators (pPTs), which counter exchange various phosphorylated carbon compounds for inorganic phosphate thereby regulating the interactions of plastid metabolism with that of the cytosol [22]. The apicoplast outer membrane pPTs (known as *Pf*oTPT in *P*. *falciparum* and *Tg*ATP1 in *T*. *gondii*) have been demonstrated to have similar transport capacities and are essential [23–25], apparently because they supply apicoplasts with crucial sources of carbon, energy and reducing power by importing products of cytosolic glycolysis into the organelle [26].

Lack of a bipartite, apicoplast targeting leader on parasite pPTs was initially rather puzzling, and it was proposed that the first transmembrane domain (TMD; the proteins have 10 in total) might act as a classic signal anchor mediating insertion of the protein into the ER membrane, with subsequent transfer via vesicular traffic into the outer apicoplast membrane, that is at least transiently contiguous with the endomembrane system [12]. Several additional apicoplast outer membrane proteins have since been identified; all lack a bipartite leader but do have potential signal anchors [27–31]. Mutagenesis analysis of *Tg*ATP has confirmed that TMD1 acts as a signal anchor in *T*. *gondii* and also identified a tyrosine-based motif in the cytosolic N-terminus of the protein as necessary, but not sufficient, for apicoplast localisation [32]. Similar tyrosine-based motifs occur in the pPTs of other Apicomplexan parasites including *Pf*oTPT, and the *P*. *vivax* pPT N-terminus was able to direct *Tg*ATP1 to the apicoplast [32]. We decided to explore the targeting of the malaria parasite apicoplast pPT, *Pf*oTPT, *in vivo* to further define elements or architecture essential for targeting to the apicoplast membrane. A better understanding of targeting to the outer membrane, which cannot yet be predicted, could help identify other apicoplast transporters and membrane proteins for improved understanding of organelle function. Here we describe the roles of different combinations of *Pf*oTPT TMDs in subcellular targeting and the critical role of a tyrosine in the cytosolic N-terminus for correct apicoplast targeting, similar to the situation in *T*. *gondii*.

## Materials and Methods

### Cloning of Constructs

**Fig 1:**
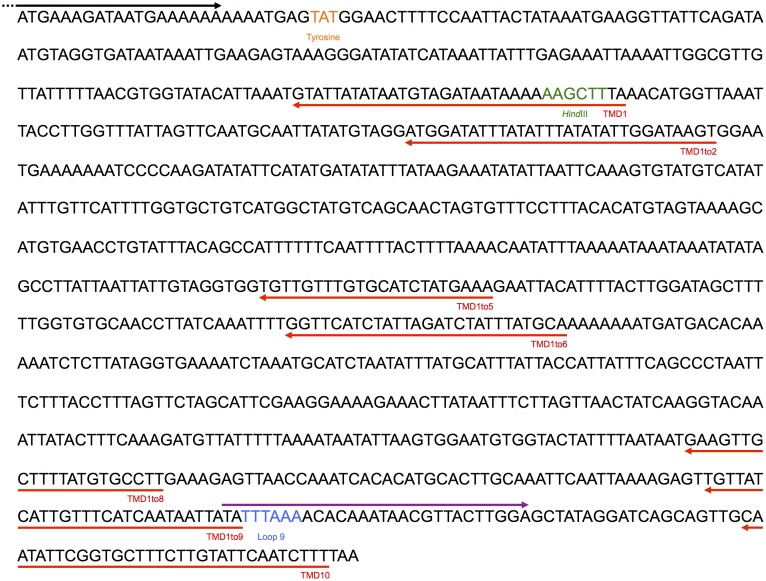
DNA sequence of the *Pf*oTPT gene from start to stop codon. The part of the forward primer found in the coding region is shown in black and was used to generate TMD1to9, TMD1to8, TMD1to6, TMD1to5, TMD1to2 and TMD1. The part of the TMD10 forward primer matching the coding region is shown in purple. Reverse primers used to amplify portions of the gene containing various TMDs are shown in red. The tyrosine codon is shown in orange, the *Hind*III cloning site is shown in green and loop 9, included in TMD1+TMD10, is shown in blue.

Truncated versions of *Pf*oTPT were amplified with the appropriate primers (Table 1 and Fig 1) from *P*. *falciparum* genomic DNA (gDNA) by polymerase chain reaction (PCR). TMD1+TMD10 was generated by fusion of TMD1 and TMD10 (containing loop 9) fragments via a *Hind*III site (Table 1 and Fig 1). The forward primer was designed such that the amplified insert would contain CACC at the 5’ end for insertion into the pENTR/D-TOPO vector (Invitrogen). The PCR reaction used Elongase (Invitrogen) and low annealing and elongation temperatures to accommodate the A/T bias in *P*. *falciparum* sequences [10].

**Table 1:** Predicted masses and primer pairs for the *Pf*oTPT transfection constructs. The tyrosine (Y) codon in synthetic *Pf*oTPT and the alanine (A) codon in Y10A are in bold. The predicted mass given for TMD10 is for the entire TMD1+TMD10 fusion protein. The *Hind*III cloning sites are underlined. A *Hind*III site is naturally found within TMD1 and one was added to the TMD10 forward primer.

Construct	Predicted mass	Forward primer	Reverse primer
Synthetic *Pf*oTPT	39.0 kDa	5’CACCATGAAAGATAACGAAAAAAAAAACGAATACGGTACGTT	5’AAAGATAGAGTATAGAAATGCTCCAAAGATGGC
Y10A	38.9 kDa	5’CACCATGAAAGATAACGAAAAAAAAAACGAAGCTGGTACG	5’AAAGATAGAGTATAGAAATGCTCCAAAGATGGC
TMD1to9	39.5 kDa	5’CACCTAAATAGAATGAAAGATAATGAAAAAA	5’TATAATTATTGATGAAACAATGATAACA
TMD1to8	36.4 kDa	5’CACCTAAATAGAATGAAAGATAATGAAAAAA	5’AAGGCACATAAAAGCAACTTC
TMD1to6	27.0 kDa	5’CACCTAAATAGAATGAAAGATAATGAAAAAA	5’TGCATAAATAGATCTAATAGATGAACC
TMD1to5	24.0 kDa	5’CACCTAAATAGAATGAAAGATAATGAAAAAA	5’TTTCATAGATGCACAAACAACA
TMD1to2	14.2 kDa	5’CACCTAAATAGAATGAAAGATAATGAAAAAA	5’ACTTATCCAATATATAAATATAAATATCCAT
TMD1	10.8 kDa	5’CACCTAAATAGAATGAAAGATAATGAAAAAA	5’TAAAGCTTTTTTATTATCTACATTATATAATAC
TMD10	10.55 kDa	5’CACCAAGCTTTATTTAAAACACAAATAACGTTACTTGGA	5’AAAGATTGAATACAAGAAAGCACCGAATATTG

The full length of *Pf*oTPT was codon optimised as previously described [24]. With this resource available to us, synthetic *Pf*oTPT was prepared with the appropriate primers (Table 1) for insertion into the *Plasmodium* transfection vector. To generate a synthetic *Pf*oTPT construct with a single point mutation (Y10A), a forward primer containing the mutation was used to amplify the codon optimised gene (Table 1).

The amplified sequences were inserted into an entry vector (pENTR^™^/D-TOPO; Invitrogen) via a TOPO reaction (Invitrogen), according to the manufacturer’s protocol. The entry vector was transformed into electrocompetent PMC103 *Escherichia coli* by electroporation. Transformed colonies were selected with 50 μg/mL kanamycin and screened by PCR with *Taq* polymerase (Invitrogen) using the gene-specific forward primer and M13 reverse primer (5’ CAGGAAACAGCTATGAC). Positive colonies were grown in culture before purification of the plasmid, according to the manufacturer’s protocol (QIAGEN). Purified entry vectors containing the insert were sequenced.

The fragment of *Pf*oTPT was transferred from the purified entry vector into an expression vector, pCHD-3/4, carrying the CRT promoter and triple hemagglutinin (HA) tag at the 3’end of the coding sequence, by the Multisite Gateway^™^ recombination system (Invitrogen), using the *att*1 and *att*2 sites in a LR reaction, according to the manufacturer’s protocol. The expression vector was transformed into *E*. *coli* cells as described above, and positive colonies were selected on their ability to grow in the presence of 100 μg/mL ampicillin. Positive colonies were screened with gene-specific primers and appropriate restriction enzyme digestions. The expression vector was then purified as above and sequenced prior to transfection.

### Transfection and Culture of Parasites

*P*. *falciparum* parasites (D10 strain) were cultured in human red blood cells at 2% haematocrit in RPMI 1640 (WEHI) supplemented with Albumax (GibcoBRL) to a final concentration of 1% and gassed with 5% CO_2_ and 1% O_2_ in N_2_ at 37°C [33]. Transfections were conducted by electroporation of ring stage parasites [34, 35] using approximately 100 μg of plasmid DNA. After electroporation, parasites were grown in the absence of drug through one 48-hour intra-erythrocytic cycle. Subsequently, WR99210 was added to a final concentration of 10 pM in the culture medium. Transformed parasites were selected based on their resistance to the antifolate WR99210 via possession of the human *dihydrofolate reductase* gene [36].

### Protein Partitioning

Infected red blood cells were lysed with 10 volumes of 0.15% saponin (Sigma-Aldrich)/0.1% bovine serum albumin (BSA; Sigma-Aldrich) and washed with phosphate buffered saline (PBS). Isolated parasites were lysed in 1% Triton X-114 (Sigma-Aldrich)/2 mM EGTA/protease inhibitor (Roche)/PBS and incubated on ice for 30 minutes [12, 37]. Cell debris was removed by centrifugation. The cloud point of Triton X-114 is approximately 20°C [12, 37], so proteins were phase partitioned by condensation by layering the supernatant onto a sucrose cushion (6% sucrose/0.06% Triton X-114/PBS) and incubating at 37°C for 3 minutes. Centrifugation resulted in the formation of three phases: the upper soluble phase, the sucrose cushion, and an insoluble pellet. This soluble phase was considered a wash, and the sucrose cushion was discarded. Partitioning was repeated on the pellet to ensure that the soluble and detergent phases were not contaminated with proteins of the wrong phase. Proteins of the phases were precipitated by acetone and analysed by Western blot.

### Western Blot Analysis

Parasites were harvested by treatment with 0.15% saponin/0.1% BSA/PBS for 10 minutes on ice. Parasites were then pelleted by centrifugation, washed with PBS and lysed in reducing sample buffer (2% β-mercaptoethanol/NUPAGE LDS Sample Buffer; Life Technologies) before being boiled for 5 minutes. Proteins were separated by SDS-PAGE [38] and transferred onto a nitrocellulose membrane (Amersham). To visualise HA-containing fragments, the membrane was blocked in western blocking buffer (5% skim milk powder/TTBS) for at least 1 hour and incubated in a monoclonal rat anti-HA antibody (Roche) diluted 1:250 in blocking buffer for 1 hour. For detection of Hsp60, anti-Hsp60 (kind gift from T. Lithgow and R. Waller) was used at a 1:1000 dilution. The membrane was then washed several times with TTBS before incubating for 45 minutes in rabbit anti-rat horseradish peroxidase-conjugated antibody (DAKO) diluted 1:1000 in blocking buffer. After repeating the wash steps, protein bands were detected on X-ray film or on a ChemiDoc (BioRad) using SuperSignal West Pico Chemiluminescent Substrate (Thermo Scientific), according to the manufacturer’s protocol.

### Immunofluorescence Assays

Cells were harvested by centrifugation, washed with PBS and fixed with 4% paraformaldehyde/0.005%-0.0075% glutaraldehyde (ProSciTech) in PBS for 20 minutes. Fixed cells were washed in PBS and subsequently permeabilised with 0.1% Triton X-100 (Fisher Scientific) in PBS for 10 minutes before being blocked with 3% BSA in PBS for at least 30 minutes. Cells were incubated with a monoclonal rat anti-HA antibody (Roche; 1:250 dilution) and the apicoplast marker acyl carrier protein (ACP) anti-serum at a 1:500 dilution, the plasma membrane marker anti-*Pf*NT1 antibody (Malaria Research and Reference Resource Centre, NIH [39]) at a 1:400 dilution or the ER marker anti-BiP antibody (Malaria Research and Reference Resource Centre, NIH [40]) at a 1:1000 dilution, in the blocking media for 1 hour at room temperature or overnight at 4°C. Samples were then washed well with PBS and incubated with goat anti-rat antibody Alexa Fluor 488 and goat anti-rabbit antibody Alexa Fluor 546 (Molecular Probes; 1:750–5000 dilution) for 1 hour. After washing with PBS, cells were incubated in Hoechst 33342 (Life Technologies; 1:10,000 dilution) for 20 minutes. Cells were adhered to 0.1% polyethylenimine (Sigma-Aldrich)-coated coverslips with a drop of 0.01% DABCO (Sigma-Aldrich) in 50% glycerol. Alternatively, immunofluorescence assays were performed on cells bound to coverslips treated with 0.5 mg/mL Concanavalin A (Sigma-Aldrich) as previously described [41]. Samples were then sealed with VALAP (Vaseline: lanolin: paraffin; 1:1:1, by weight). Control cells were incubated with secondary antibody only.

### Confocal Microscopy

Cells were observed using an inverted Leica DMIRB-TCS SP 2 confocal microscope, with PL APO 63X/1.4 λ_BL_ oil objective. Transmission images were captured using differential interference contrast optics.

## Results and Discussion

To investigate signals responsible for targeting proteins into the outer apicoplast membrane in the human malaria parasite *P*. *falciparum*, we generated a series of episomally expressed constructs incorporating different combinations of TMDs and connecting loops from the *Pf*oTPT protein, each with a C-terminal triple HA tag. Constructs were driven by the *Pf*CRT promoter, providing low expression peaking during the ring stage [12]. This stage has distinct, punctate apicoplasts [42] allowing unequivocal assessment of targeting by immunofluorescence. As previously reported [12], expression of the full gene with a C-terminal tag results in accumulation in the apicoplast and the protein is membrane integrated.

**Fig 2:**
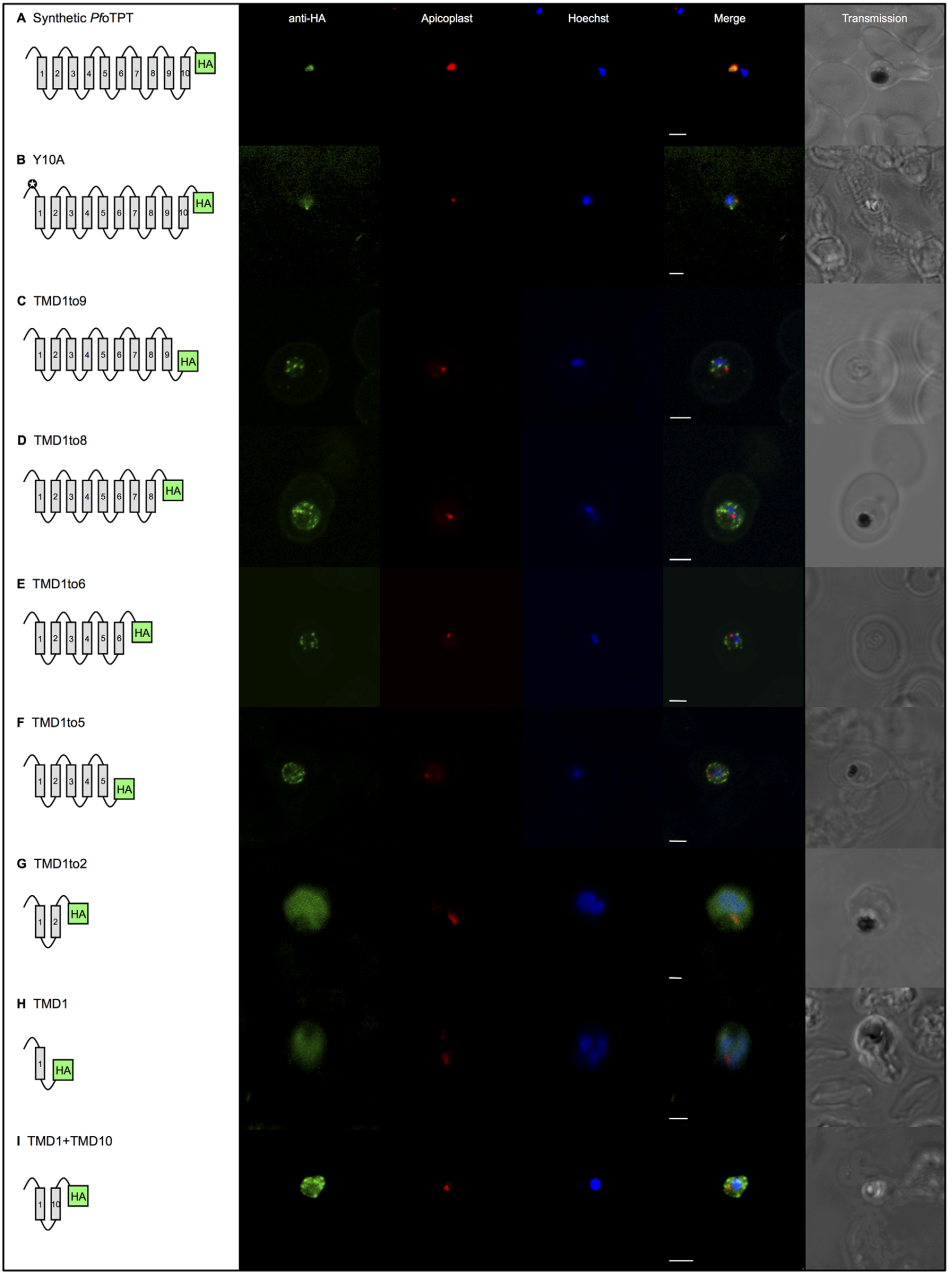
Gene expression constructs defining elements of *Pf*oTPT essential for targeting to the outer membrane of the apicoplast. TMDs are represented by numbered boxes and are joined by loops. All *Pf*oTPT constructs are episomally expressed under the *Pf*CRT promoter and tagged with triple HA at the C-terminus detected with anti-HA and secondary antibody conjugated to FITC (green) in parasites within erythrocytes. Co-localisation of the apicoplast using antisera against apicoplast stromal marker, ACP, is shown in red. Nuclei are stained with Hoechst (blue), and transmitted light images of the parasites within their host red blood cell are shown on the right. Scale bars = 2μm. A. Full length, synthetic *Pf*oTPT co-localises with ACP. B. Point mutation of tyrosine residue at position 10 (✪) in the synthetic *Pf*oTPT (Y10A) abrogates targeting to the apicoplast showing no co-localisation with the apicoplast marker. C. Removal of TMD 10 (TMD1to9) abrogates targeting to the apicoplast showing no co-localisation with the apicoplast marker. D. Removal of TMDs 9 and 10 (TMD1to8) abrogates targeting to the apicoplast showing no co-localisation with the apicoplast marker. E. Removal of TMDs 7, 8, 9 and 10 (TMD1to6) abrogates targeting to the apicoplast showing no co-localisation with the apicoplast marker. F. Removal of TMDs 6, 7, 8, 9 and 10 (TMD1to5) abrogates targeting to the apicoplast showing no co-localisation with the apicoplast marker. G. Removal of TMDs 3, 4, 5, 6, 7, 8, 9 and 10 (TMD1to2) abrogates targeting to the apicoplast showing no co-localisation with the apicoplast marker and diffuse staining throughout the parasite. H. Removal of TMDs 2, 3, 4, 5, 6, 7, 8, 9 and 10 (TMD1) abrogates targeting to the apicoplast showing no co-localisation with the apicoplast marker and diffuse staining throughout the parasite. I. A combination of TMD1 and TMD10 (TMD1+TMD10) was not sufficient to reconstitute targeting to the apicoplast, showing no co-localisation with the apicoplast marker.

**Fig 3:**
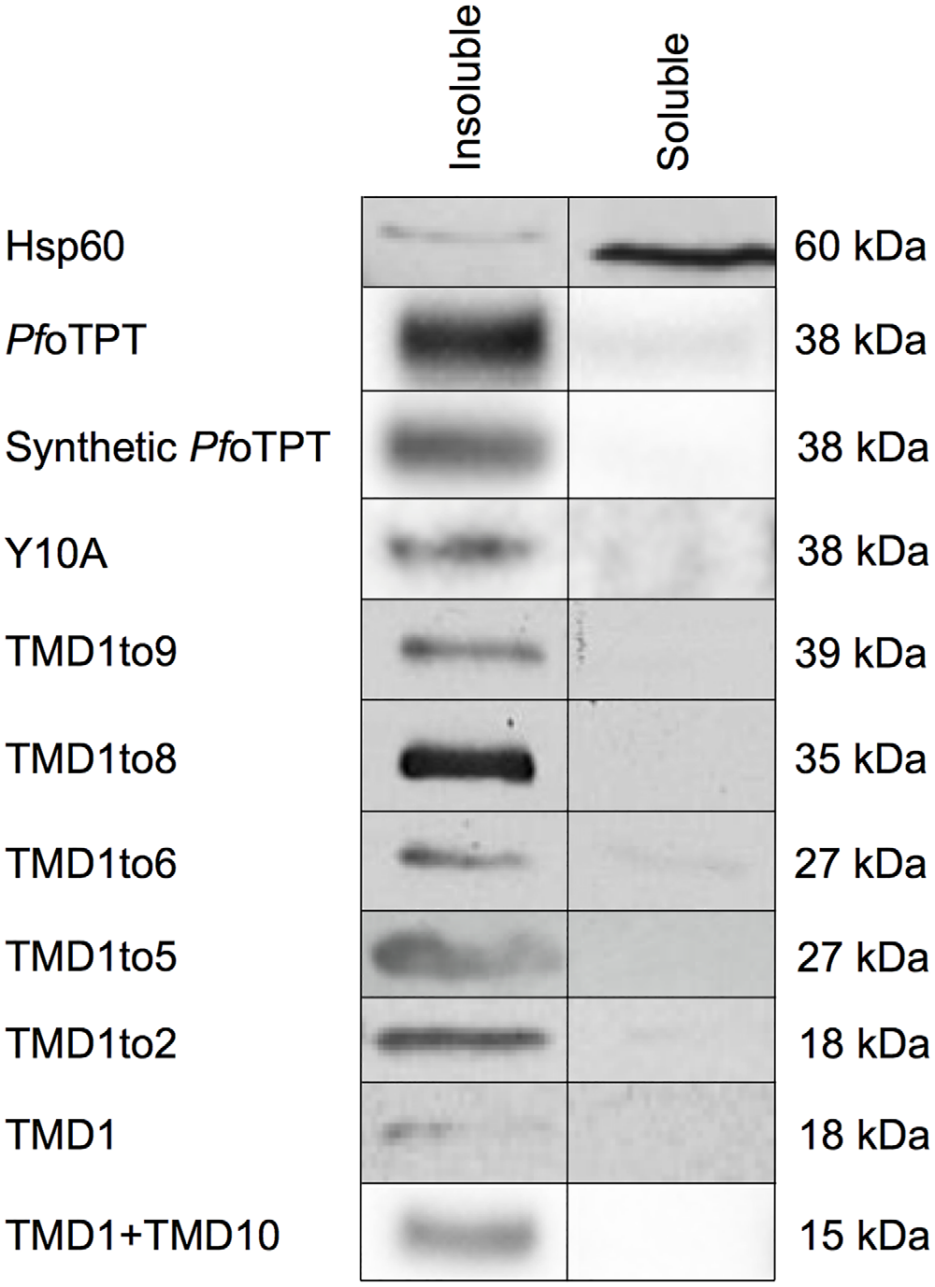
Western blots showing that all *Pf*oTPT modification constructs produce membrane bound proteins of appropriate apparent masses. The left column shows the insoluble (membrane) fraction while the right column shows the soluble (non-membrane) fractions after Triton X-114 protein partitioning. The apicoplast stromal protein Hsp60 [44] is loaded as a soluble protein control, and *Pf*oTPT is loaded as an insoluble protein control [12]. Hsp60 fractions were probed with anti-Hsp60 and all other fractions with anti-HA.

To avoid integration of constructs that could confound some of the planned experiments, we used a synthetic gene with different codon usage (available to us from previous work [24]). Expression of the full length, synthetic, HA-tagged gene showed faithful targeting to the apicoplast (Fig 2A) and Triton X-114 phase partitioning demonstrated that the protein is lodged in the apicoplast membrane (Fig 3). We then made a point mutation within this construct to change the tyrosine residue at position 10 in the cytosolic N-terminus to alanine (Y10A; Table 1). This single point mutation drastically altered targeting of the protein with none visible within the apicoplast and the overwhelming majority of protein accumulating outside the apicoplast (Fig 2B). The Y10A construct remained membrane resident (Fig 3), and is apparently located in both the plasma membrane on the basis of co-localisation with the plasma membrane marker, *Pf*NT1 (S1A Fig), a nucleoside transporter shown to reside in the parasite plasma membrane [43], and the ER on the basis of co-localisation with BiP (S2A Fig), a member of the heat shock protein family (*Pf*HSP70) resident in the ER [40]. These results corroborate identification of this tyrosine motif as essential for apicoplast outer membrane targeting of *Tg*ATP1, the orthologous protein in *T*. *gondii* [32].

Although these studies clearly identify the Y10A motif as necessary for apicoplast outer membrane targeting, the motif alone is apparently not sufficient to direct any membrane protein to this destination as previous fusion of the N-terminus (containing the crucial tyrosine motif) to a nucleoside sugar transporter (*Tg*NST1) did not confer apicoplast targeting in *T*. *gondii* [32]. To explore the possibility that another domain (or domains) in these proteins is required for outer apicoplast membrane targeting, we created constructs from *Pf*oTPT with various combinations of loops and TMDs and with C-terminal epitope tags (Fig 2). These constructs, which use the native *Pf*oTPT DNA sequence, showed no tendency to integrate; likely because integrations would truncate the *Pf*oTPT protein, which is essential in *P*. *berghei* [25] and thus likely to be essential in *P*. *falciparum*. Since there is no need for processing of *Pf*oTPT for correct targeting [12], our strategy should yield information on what portions of the protein are necessary for apicoplast targeting. Such a truncation approach has also successfully dissected the ER targeting signals of ryanodine receptors in mammalian cells [45] and facilitated the examinations of the targeting requirements and topology of plant vacuolar antiporters [46] and mammalian ATP-binding cassette (ABC) transporters [47].

Our first deletion construct (TMD1to9) removed TMD 10, which abrogated apicoplast targeting (Fig 2C). Even though this construct contains almost the entire *Pf*oTPT protein, it did not target to the apicoplast as shown by the lack of co-localisation with ACP (Fig 2C). TMD1to9 also did not target to the plasma membrane on the basis of no co-localisation with nucleoside transporter, *Pf*NT1 (S1B Fig). Rather, TMD1to9 exhibited peri-nuclear staining typical of ER localisation in *Plasmodium* spp., confirmed by co-localisation with BiP (S2B Fig).

Four other constructs, each lacking further C-terminal TMDs (TMD1to8, TMD1to6, TMD 1to5, and TMD1to2), also failed to target to the apicoplast on the basis of no detectable co-localisation with ACP (Fig 2D–2G). Nevertheless, all these deletion constructs remained membrane resident (Fig 3) and tended to localise to the ER and/or the plasma membrane on the basis of co-localisation with BiP and/or *Pf*NT1 (S1C–S1F and S2C Figs) (BiP co-localisation data not shown for TMD1to8, TMD1to6, TMD1to5). We conclude that truncations of the *Pf*oTPT C-terminus abrogates apicoplast targeting, which could suggest that the full complement of 10 TMDs, and/or some element in the C-terminal tail, are essential for apicoplast targeting.

The minimal construct tested was TMD1, and it did not localise to the apicoplast (Fig 2H) but instead showed ER localisation (S2D Fig) and plasma membrane localisation (S1G Fig) on the basis of co-localisation with BiP and *Pf*NT1, respectively. TMD1 is primarily a membrane located protein (Fig 3), and along with the other C-terminal deletion constructs (above), these localisations support the hypothesis [12] that TMD1 probably acts as a recessed signal anchor directing *Pf*oTPT into the endomembrane, which has also been confirmed for *Tg*ATP1 [32].

Our final construct, TMD1+TMD10, sought to bring together three of the elements identified in this study as apparently being essential for apicoplast outer membrane targeting (the tyrosine motif in the cytosolic N-terminus, TMD1, and TMD10, and the cytosolic C-terminus), as a potentially minimal construct that might be sufficient to target a protein into the apicoplast outer membrane. However, this construct clearly did not co-localise with ACP (Fig 2I), instead appearing to accumulate in the parasite endomembrane (S2E Fig) and plasma membrane (S1H Fig). TMD1+TMD10 is exclusively a membrane located protein (Fig 3); its orientation is unknown but assumed to have the termini projecting into the cytosol on the basis of the positively charged N-terminus and the even number of TMDs.

An alternative explanation for the loss of targeting in these truncated constructs is altered orientation of the proteins in the membrane. We previously showed that *Pf*oTPT resides in the outer apicoplast membrane with its termini facing the cytosol [12], likely because of the positive charges on the N-terminus and the even number of TMDs. The impact of protein orientation on apicoplast targeting remains unexplored. Unfortunately, determining the membrane orientation of artificial constructs in the endomembrane system is technically challenging and beyond the scope of this study. Therefore, it remains possible that the deletions of TMDs in *Pf*oTPT alter membrane topology causing mis-localisation. It is reasonable, however, to assume that constructs including the positive N-terminus and an even number of TMDs (e.g. TMD1to8, TMD1to6, TMD1to2, TMD1+TMD10) would adopt the same configuration as full length *Pf*oTPT, but they still all failed to target to the apicoplast. From this observation, we tentatively conclude that TMD symmetry is not sufficient for apicoplast targeting in these constructs.

All constructs migrated in SDS-PAGE gels at appropriate apparent masses and were membrane resident (Fig 3).

## Conclusions

The localisation of *Pf*oTPT to the apicoplast heralded a new paradigm in apicoplast targeting [12, 27]. *Pf*oTPT lacks the classical bipartite leader necessary for targeting proteins to the apicoplast stroma [11] and was hypothesised to utilise a previously uncharacterised route that could direct proteins into the outermost apicoplast membranes [12]. Mullin et al. [12] proposed that TMD1 of *Pf*oTPT is a recessed signal anchor that directs the protein into the endomembrane and that another feature (or features) in the protein must be responsible for directing and lodging the protein in the outermost apicoplast membrane. Here we demonstrated that the first TMD of *Pf*oTPT is indeed sufficient for committing the protein into the endomembrane system in which the apicoplast is positioned, but is insufficient for apicoplast targeting. This is consistent with the hypothesis that TMD1 is a recessed signal anchor [12], as previously shown in *T*. *gondii* [32].

We also confirm that a tyrosine residue, present in the N-termini of all apicomplexan homologues of *Pf*oTPT and crucial for *T*. *gondii* outer apicoplast membrane targeting [32], is also essential for correct targeting in *P*. *falciparum*. However, the lack of plastid targeting by TMD1 alone, even though it includes the critical tyrosine residue in the N-terminus, tells us that one or more other features of the protein are necessary for apicoplast outer membrane targeting. Orientation and protein topology may be important factors for correct apicoplast localisation. The C-terminal deletion constructs perhaps indicate that TMD10 and/or the C-terminus are critical for faithful targeting, but an attempt to constitute these elements into a minimal apicoplast outer membrane-targeting module (TMD1+TMD10) was unsuccessful. In summary, TMD1 is likely to act as a signal anchor to direct *Pf*oTPT into the endomembrane system. A tyrosine in the N-terminus is necessary for apicoplast outer membrane targeting, and loss of TMD10 and the C-terminus perturbs targeting, but a minimal combination of these three elements is not sufficient to reconstruct targeting. Thus, an as yet unidentified element (or elements), or perhaps even protein orientation in the membrane, is required for targeting to the outer apicoplast membrane.

## Supporting Information

S1 Fig Gene expression constructs not targeted to the apicoplast now co-localised with the plasma membrane. TMDs are represented by boxes and are joined by loops. All *Pf*oTPT constructs are episomally expressed under the *Pf*CRT promoter and tagged with triple HA at the C-terminus detected with anti-HA and secondary antibody conjugated to FITC (green). The parasite plasma membrane is detected with anti-*Pf*NTP1 (red). Nuclei are stained with Hoechst (blue), and transmitted light images of the parasites within their host red blood cell are shown at right. Scale bars = 2μm. A. Point mutation of tyrosine residue at position 10 (✪) in the synthetic *Pf*oTPT (Y10A) relocates protein from the apicoplast (Fig 2B) to the plasma membrane. B. Removal of TMD 10 (TMD1to9), which completely abrogates targeting to the apicoplast (Fig 2C), does not result in plasma membrane localisation. C. Removal of TMDs 9 and 10 (TMD1to8), which completely abrogates targeting to the apicoplast (Fig 2D), results in some protein localisation to the plasma membrane. D. Removal of TMDs 7, 8, 9 and 10 (TMD1to6), which completely abrogates targeting to the apicoplast (Fig 2E), results in localisation of the protein that mostly overlaps with the parasite plasma membrane marker, *Pf*NT1. Note that the erythrocyte is infected with three separate parasites. E. Removal of TMDs 6, 7, 8, 9 and 10 (TMD1to5), which completely abrogates targeting to the apicoplast (Fig 2F), results in localisation of the protein that mostly overlaps with the parasite plasma membrane marker, *Pf*NT1. Note that the erythrocyte is infected with two separate parasites. F. Removal of TMDs 3, 4, 5, 6, 7, 8, 9 and 10 (TMD1to2), which completely abrogates targeting to the apicoplast (Fig 2G), results in some re-localisation of the protein to the plasma membrane. G. Removal of TMDs 2, 3, 4, 5, 6, 7, 8, 9 and 10 (TMD1), which completely abrogates targeting to the apicoplast (Fig 2H), results in some localisation of the protein to the plasma membrane. H. Recombining TMD1 with TMD10 (TMD1+10) was unable to reconstitute apicoplast targeting (Fig 2I) and resulted in some plasma membrane targeting.
(TIFF)

S2 Fig Select gene expression constructs not targeted to the apicoplast are now co-localised with the endoplasmic reticulum. TMDs are represented by boxes and are joined by loops. All *Pf*oTPT constructs are episomally expressed under the *Pf*CRT promoter and tagged with triple HA at the C-terminus detected with anti-HA and secondary antibody conjugated to FITC (green). The parasite ER is detected with anti-BiP (red). Nuclei are stained with Hoechst (blue), and transmitted light images of the parasites within their host red blood cell are shown at right. Scale bars = 2μm. A. Point mutation of tyrosine residue at position 10 (✪) in the synthetic *Pf*oTPT (Y10A) relocates protein from the apicoplast (Fig 2B) to the ER, as well as the plasma membrane (S1A Fig). B. Removal of TMD 10 (TMD1to9), which completely abrogates targeting to the apicoplast (Fig 2C), results in perinuclear localisation of the protein that almost entirely overlaps with the ER marker, BiP. C. Removal of TMDs 3, 4, 5, 6, 7, 8, 9 and 10 (TMD1to2), which completely abrogates targeting to the apicoplast (Fig 2G), results in re-localisation of most of the protein to the ER. D. Removal of TMDs 2, 3, 4, 5, 6, 7, 8, 9 and 10 (TMD1), which completely abrogates targeting to the apicoplast (Fig 2H), primarily results in localisation of most of the protein to the ER. E. Recombining TMD1 with TMD10 (TMD1+10) was unable to reconstitute apicoplast targeting (Fig 2I) and resulted in some perinuclear ER targeting, plus some parasite plasma membrane targeting (S1H Fig).
(TIFF)
