# Correction: Targeting of a Transporter to the Outer Apicoplast Membrane in the Human Malaria Parasite *Plasmodium falciparum *

**DOI:** 10.1371/journal.pone.0161420

**Published:** 2016-08-22

**Authors:** The PLOS ONE Staff

There is an error in Table 1. The codons should be shown in bold. Please view the correct table here. The publisher apologizes for the error.

**Table 1:** Predicted masses and primer pairs for the *Pf*oTPT transfection constructs. The tyrosine (Y) codon in synthetic *Pf*oTPT and the alanine (A) codon in Y10A are in bold. The predicted mass given for TMD10 is for the entire TMD1+TMD10 fusion protein. The *Hind*III cloning sites are underlined. A *Hind*III site is naturally found within TMD1 and one was added to the TMD10 forward primer.

Construct	Predicted mass	Forward primer	Reverse primer
Synthetic *Pf*oTPT	39.0 kDa	5’CACCATGAAAGATAACGAAAAAAAAAACGAA**TAC**GGTACGTT	5’AAAGATAGAGTATAGAAATGCTCCAAAGATGGC
Y10A	38.9 kDa	5’CACCATGAAAGATAACGAAAAAAAAAACGAA**GCT**GGTACG	5’AAAGATAGAGTATAGAAATGCTCCAAAGATGGC
TMD1to9	39.5 kDa	5’CACCTAAATAGAATGAAAGATAATGAAAAAA	5’TATAATTATTGATGAAACAATGATAACA
TMD1to8	36.4 kDa	5’CACCTAAATAGAATGAAAGATAATGAAAAAA	5’AAGGCACATAAAAGCAACTTC
TMD1to6	27.0 kDa	5’CACCTAAATAGAATGAAAGATAATGAAAAAA	5’TGCATAAATAGATCTAATAGATGAACC
TMD1to5	24.0 kDa	5’CACCTAAATAGAATGAAAGATAATGAAAAAA	5’TTTCATAGATGCACAAACAACA
TMD1to2	14.2 kDa	5’CACCTAAATAGAATGAAAGATAATGAAAAAA	5’ACTTATCCAATATATAAATATAAATATCCAT
TMD1	10.8 kDa	5’CACCTAAATAGAATGAAAGATAATGAAAAAA	5’TAAAGCTTTTTTATTATCTACATTATATAATAC
TMD10	10.55 kDa	5’CACCAAGCTTTATTTAAAACACAAATAACGTTACTTGGA	5’AAAGATTGAATACAAGAAAGCACCGAATATTG
