# Supplementary figures and images for: Targeting of a Transporter to the Outer Apicoplast Membrane in the Human Malaria Parasite Plasmodium falciparum
Source: PLoS One. 2016 Jul 21;11(7):e0159603. doi: 10.1371/journal.pone.0159603 (PMC4956234; doi:10.1371/journal.pone.0159603)

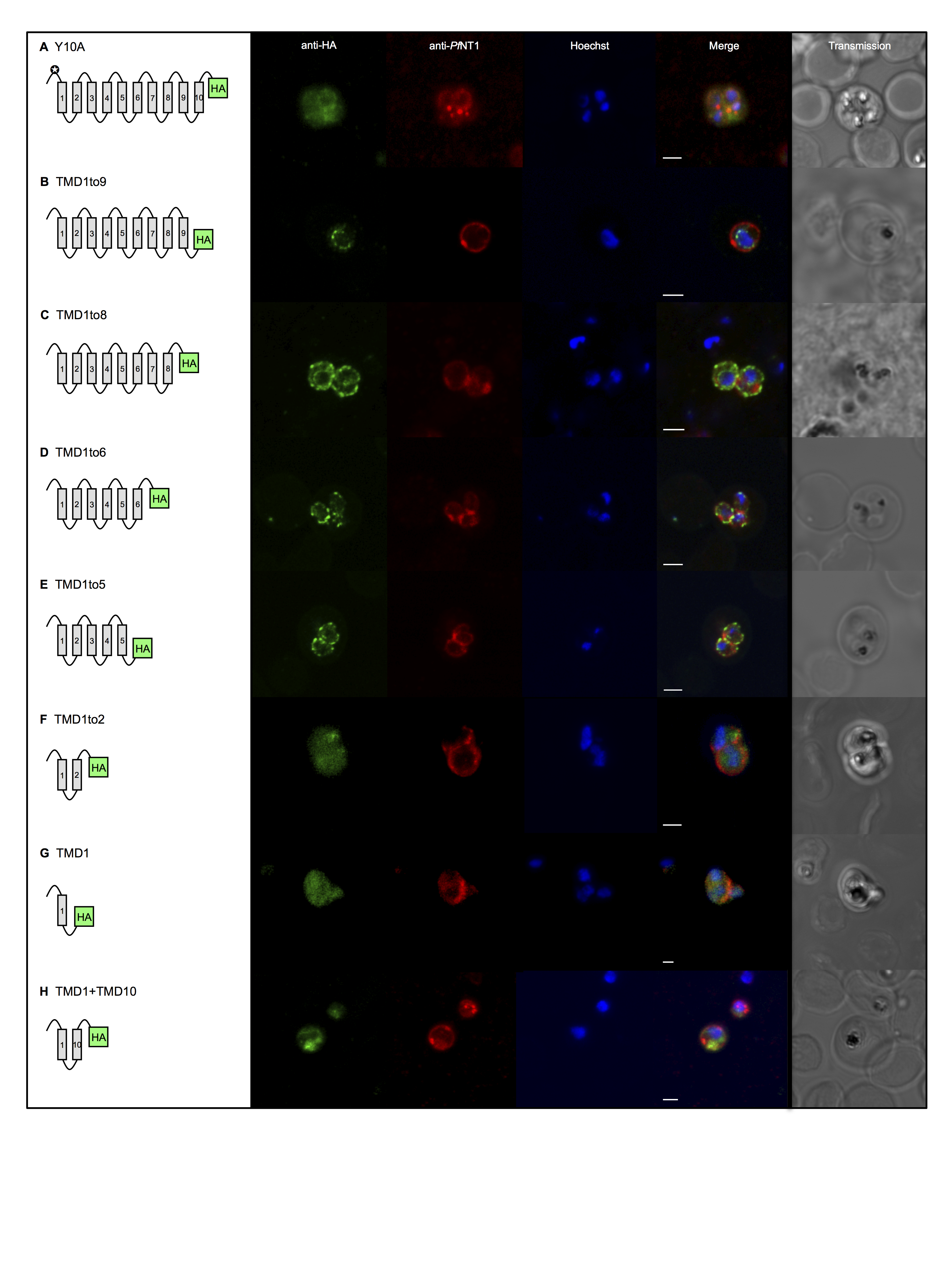

Supplement: S1 Fig — TMDs are represented by boxes and are joined by loops. All PfoTPT constructs are episomally expressed under the PfCRT promoter and tagged with triple HA at the C-terminus detected with anti-HA and secondary antibody conjugated to FITC (green). The parasite plasma membrane is detected with anti-PfNTP1 (red). Nuclei are stained with Hoechst (blue), and transmitted light images of the parasites within their host red blood cell are shown at right. Scale bars = 2μm. A. Point mutation of tyrosine residue at position 10 (✪) in the synthetic PfoTPT (Y10A) relocates protein from the apicoplast (Fig 2B) to the plasma membrane. B. Removal of TMD 10 (TMD1to9), which completely abrogates targeting to the apicoplast (Fig 2C), does not result in plasma membrane localisation. C. Removal of TMDs 9 and 10 (TMD1to8), which completely abrogates targeting to the apicoplast (Fig 2D), results in some protein localisation to the plasma membrane. D. Removal of TMDs 7, 8, 9 and 10 (TMD1to6), which completely abrogates targeting to the apicoplast (Fig 2E), results in localisation of the protein that mostly overlaps with the parasite plasma membrane marker, PfNT1. Note that the erythrocyte is infected with three separate parasites. E. Removal of TMDs 6, 7, 8, 9 and 10 (TMD1to5), which completely abrogates targeting to the apicoplast (Fig 2F), results in localisation of the protein that mostly overlaps with the parasite plasma membrane marker, PfNT1. Note that the erythrocyte is infected with two separate parasites. F. Removal of TMDs 3, 4, 5, 6, 7, 8, 9 and 10 (TMD1to2), which completely abrogates targeting to the apicoplast (Fig 2G), results in some re-localisation of the protein to the plasma membrane. G. Removal of TMDs 2, 3, 4, 5, 6, 7, 8, 9 and 10 (TMD1), which completely abrogates targeting to the apicoplast (Fig 2H), results in some localisation of the protein to the plasma membrane. H. Recombining TMD1 with TMD10 (TMD1+10) was unable to reconstitute apicoplast targeting (Fig 2I) and re [file pone.0159603.s001.tiff]

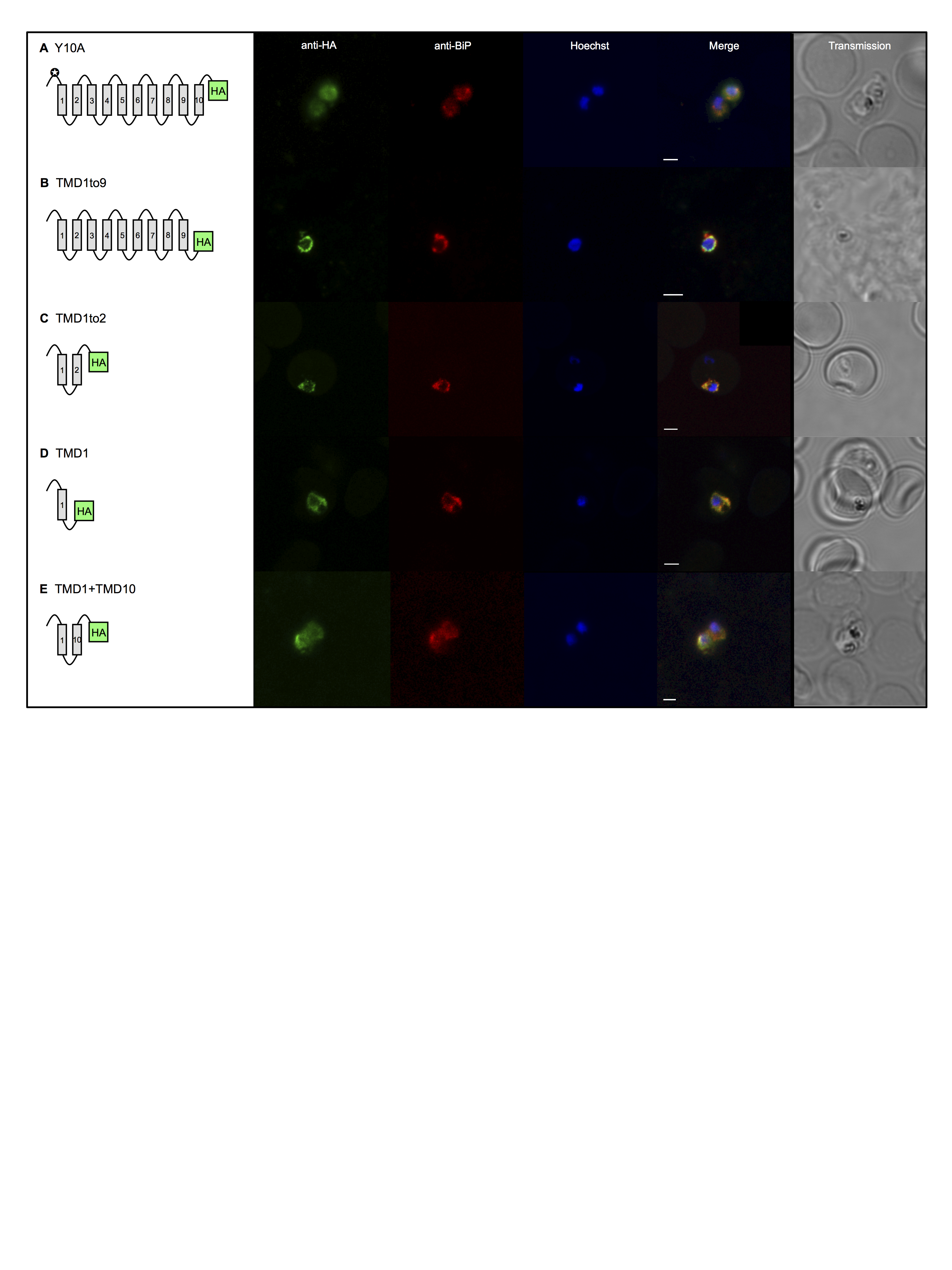

Supplement: S2 Fig — TMDs are represented by boxes and are joined by loops. All PfoTPT constructs are episomally expressed under the PfCRT promoter and tagged with triple HA at the C-terminus detected with anti-HA and secondary antibody conjugated to FITC (green). The parasite ER is detected with anti-BiP (red). Nuclei are stained with Hoechst (blue), and transmitted light images of the parasites within their host red blood cell are shown at right. Scale bars = 2μm. A. Point mutation of tyrosine residue at position 10 (✪) in the synthetic PfoTPT (Y10A) relocates protein from the apicoplast (Fig 2B) to the ER, as well as the plasma membrane (S1A Fig). B. Removal of TMD 10 (TMD1to9), which completely abrogates targeting to the apicoplast (Fig 2C), results in perinuclear localisation of the protein that almost entirely overlaps with the ER marker, BiP. C. Removal of TMDs 3, 4, 5, 6, 7, 8, 9 and 10 (TMD1to2), which completely abrogates targeting to the apicoplast (Fig 2G), results in re-localisation of most of the protein to the ER. D. Removal of TMDs 2, 3, 4, 5, 6, 7, 8, 9 and 10 (TMD1), which completely abrogates targeting to the apicoplast (Fig 2H), primarily results in localisation of most of the protein to the ER. E. Recombining TMD1 with TMD10 (TMD1+10) was unable to reconstitute apicoplast targeting (Fig 2I) and resulted in some perinuclear ER targeting, plus some parasite plasma membrane targeting (S1H Fig). (TIFF) [file pone.0159603.s002.tiff]
